# Preoperative prediction of the lymphovascular tumor thrombus of colorectal cancer with the iodine concentrations from dual-energy spectral CT

**DOI:** 10.1186/s12880-023-01060-z

**Published:** 2023-08-03

**Authors:** Xiang Yuan, Xin Quan, Xiao-ling Che, Lu-Lu Xu, Chun-mei Yang, Xiao-di Zhang, Jian Shu

**Affiliations:** 1 Department of Radiology, The Affiliated Hospital of Southwest Medical University, No.25 taiping street, 64600 Luzhou, China; 2 Philips Healthcare, 610041 Chengdu, Sichuan China

**Keywords:** Dual-Energy spectral CT, Lymphovascular tumor thrombus, Colorectal cancer, Iodine concentrations

## Abstract

**Background:**

The aim of this study was to explore application value of iodine concentration from dual-energy spectral computed tomography (DESCT) in preoperative prediction of lymphovascular tumor thrombus in patients with colorectal cancer (CRC).

**Methods:**

We finally retrospectively analyzed 50 patients with CRC who underwent abdominal DESCT before receiving any preoperative treatment and underwent surgery to obtain pathological specimens which were stained with hematoxylin-eosin (HE) staining. According to the presence of cancer cell nests in blood vessels and lymphatic vessels, the subjects were divided into the positive group and negative group of lymphovascular tumor thrombus. Two radiologists independently measured the normalized iodine concentration (NIC) values, effective atomic number (Zeff) and CT values of virtual monochromatic images (VMIs) at 40–90 keV of the primary tumors in the arterial phase (AP) and venous phase (VP). Used SPSS 17.0 to calculate the receiver operating characteristic (ROC) curve to evaluate diagnostic value.

**Results:**

The patients were divided into lymphovascular tumor thrombus positive group(n = 16) and negative group(n = 34). The values of NIC-AP and NIC-VP in the positive group were 0.17 ± 0.09, 0.51 ± 0.13, respectively. And those in the negative group were 0.15 ± 0.06, 0.43 ± 0.12, respectively. There was significant difference in NIC-VP value between the two groups (p = 0.039), but there was no significant difference in NIC-AP value (p = 0.423). The optimal threshold value of NIC-VP value for diagnosis of lymphovascular tumor thrombus was 0.364. The sensitivity was 68.8% and the specificity was 67.6%.

**Conclusions:**

The NIC-VP value of DESCT can be used to predict the presence or absence of the lymphovascular tumor thrombus in CRC patients before operation, which is helpful to select the best treatment scheme and evaluate its prognosis.

## Background

Colorectal cancer has become one of the main public health issues due to the increased morbidity and mortality rate year after year. And the 5-year survival rate of CRC patients is 30% in developing countrieswhile more than 60% in the most developed countries (eg, in Europe, North America), which has a significant discrepancy in different countries. CRC patients often have already metastasized by the time they are diagnosed. Although the therapeutic schedule and effect have obviously improved in recent years, CRC patients with distant metastasis or lymph node metastases still have a poor prognosis [1–3].

The lymphovascular tumor thrombus was found that it was having correlation of the lymph node metastasis (LNM), relapse, distant metastasis and poor prognosis in patients with CRC [4, 5]. So accurate assessment of lymphovascular tumor thrombus can provide valuable information for the treatment and prognosis of patients with CRC. Traditional method of detecting lymphovascular tumor thrombus is hematoxylin-eosin staining. When the thrombus formed by malignant tumor cells is found in the blood vessels or lymphatic vessels of pathological specimens under the microscope after staining, it can be recognized as positive of lymphovascular tumor thrombus. But it is difficult to reach a unified standard in this process, so it is easily trigger to false negative results. So it will be very beneficial to develop a new method to evaluate lymphovascular tumor thrombus more accurately in CRC patients.

Dual-energy spectral computed tomography is an emerging imaging technique in recent years. Compared with traditional CT, DESCT can provide multiple quantitative parameters, such as the normalized iodine concentration values, effective atomic number and monochromatic CT value at each energy levels (40–200 keV) [6]. A large number of studies have shown that DESCT can be used to evaluate the CRC grading, regional LNM and microsatellite instability (MSI) status [7–10]. But the predictive ability of DESCT for lymphovascular tumor thrombus in CRC has not been verified. Therefore, the purpose of this study is to investigate the relationship between the quantitative parameters of DESCT and lymphovascular tumor thrombus.

## Methods

### Patients

**Fig. 1:**
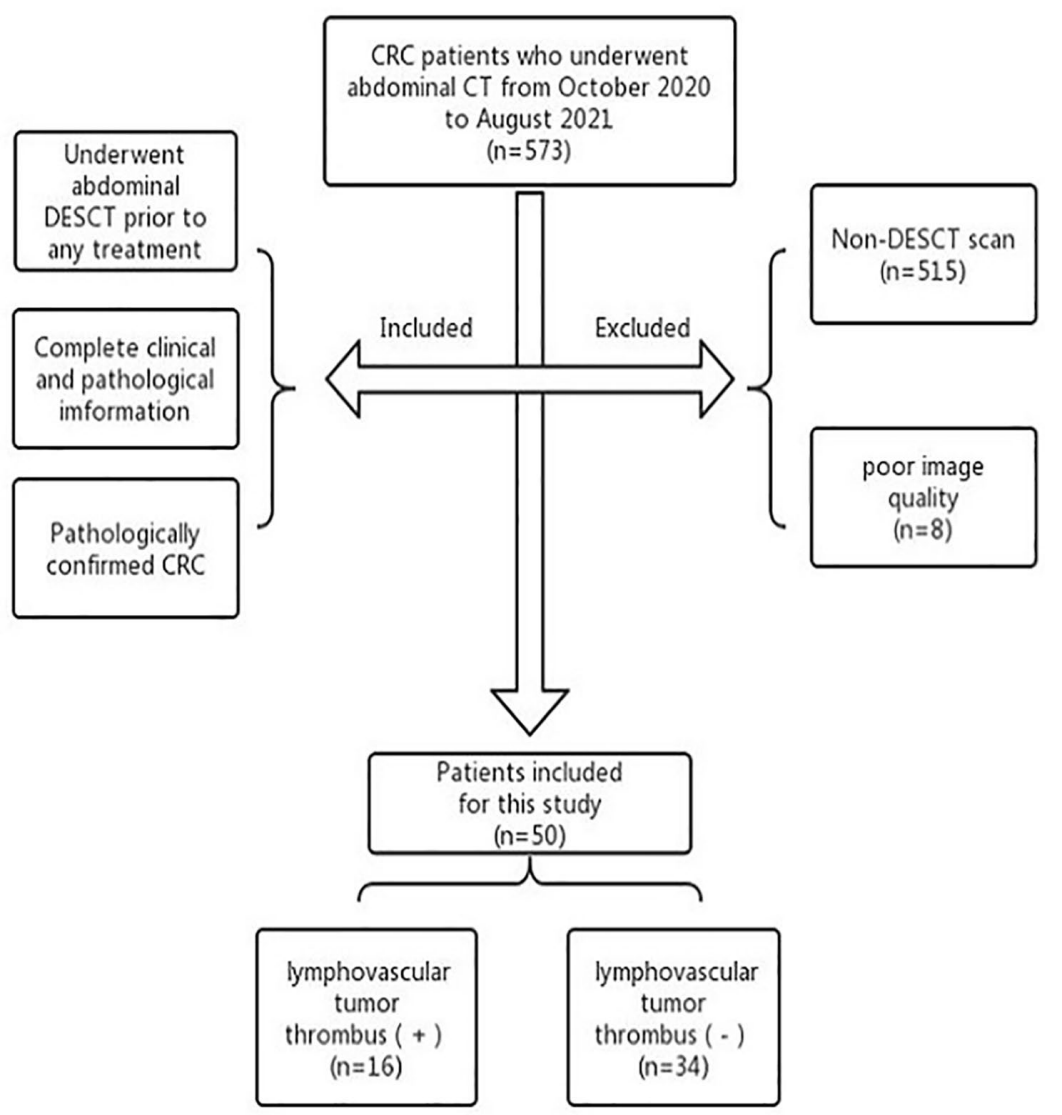
The diagram of patient recruitment Note: DESCT, dual-energy spectral CT;CRC,colorectal cancer

This study was approved by the Clinical Trial Ethics Committee of Affiliated Hospital of Southwest Medical University, and the requirement for informed consent was waived. All recruitment procedures,exclusion criteria and inclusion criteria are shown in Fig. 1. We retrospectively analyzed 573 patients with pathologically confirmed CRC from October 2020 to August 2021. In these patients, 58 patients underwent abdominal DESCT prior to any treatment. And among 58 patients, 8 patients were excluded because of poor image quality. Thus,50 patients ultimately met the inclusion criteria for this study. Clinical information was collected, including age and sex. According to the results of HE staining, the patients were divided into positive group(n = 16) and negative group(n = 34) of lymphovascular tumor thrombus. According to the pathological results, the patients were divided into LN metastasis(+) (n = 23) and LN metastasis(-) (n = 27), Peripheral nerve invasion(+) (n = 9) and Peripheral nerve invasion(-) (n = 41), Poorly Differentiated(n = 42) and Well Differentiated(n = 8) .

### Dual-energy spectral CT

Compared with traditional single-energy CT, DESCT improves the quantification of materials and the characterization of tissues, allowing for the separation of X-ray spectrum without changing the tube kilovolt peak or any other scanning parameters [11]. And it provides spectral information by using a single X-ray tube and two predefined detector layers, these two detector layers decompose the X-ray beam into high- and low-energy photons which are collected by the lower and upper detector layers, respectively [12]. And then high- and low-energy images are obtained and conventional images can be calculated by weighted summation. Spectral information can be extracted from high- and low-energy detector data.

### Imaging protocol

All patients were in supine position and bowel preparation was performed prior to examination. All patients should be fasting for 5–6 hours before the examination and drink 500ml of purified water. Conventional double-phase contrast-enhanced scan were performed by Philips IQon Spectral CT. The scanning direction was from head to feet, and the scanning range was from the top of diaphragm to the lower edge of pubic symphysis. The abdominal aorta was monitored and when it reaches 150HU, the arterial phase scan was triggered. And then the venous phase was scanned after a delay of 35s. The CTDIvol is 13.5 mGy.

The scanning parameters were as follows:a tube voltage of 120kv; automatic tube current; a rotation time of 0.5s; a section thickness of 1.0 mm; a pitch of 0.985; a detector width of 64×0.66mm; a image matrix of 512×512 pix×pix. The Iopamidol 350/300 and Omnipaque 350 injection were used in contrast-enhanced scan and the rate of injection was 3 ml/ sec; and then inject 30ml of 9% sodium chloride solution at the same injection rate after injecting the contrast agent.

### Image analysis

**Fig. 2:**
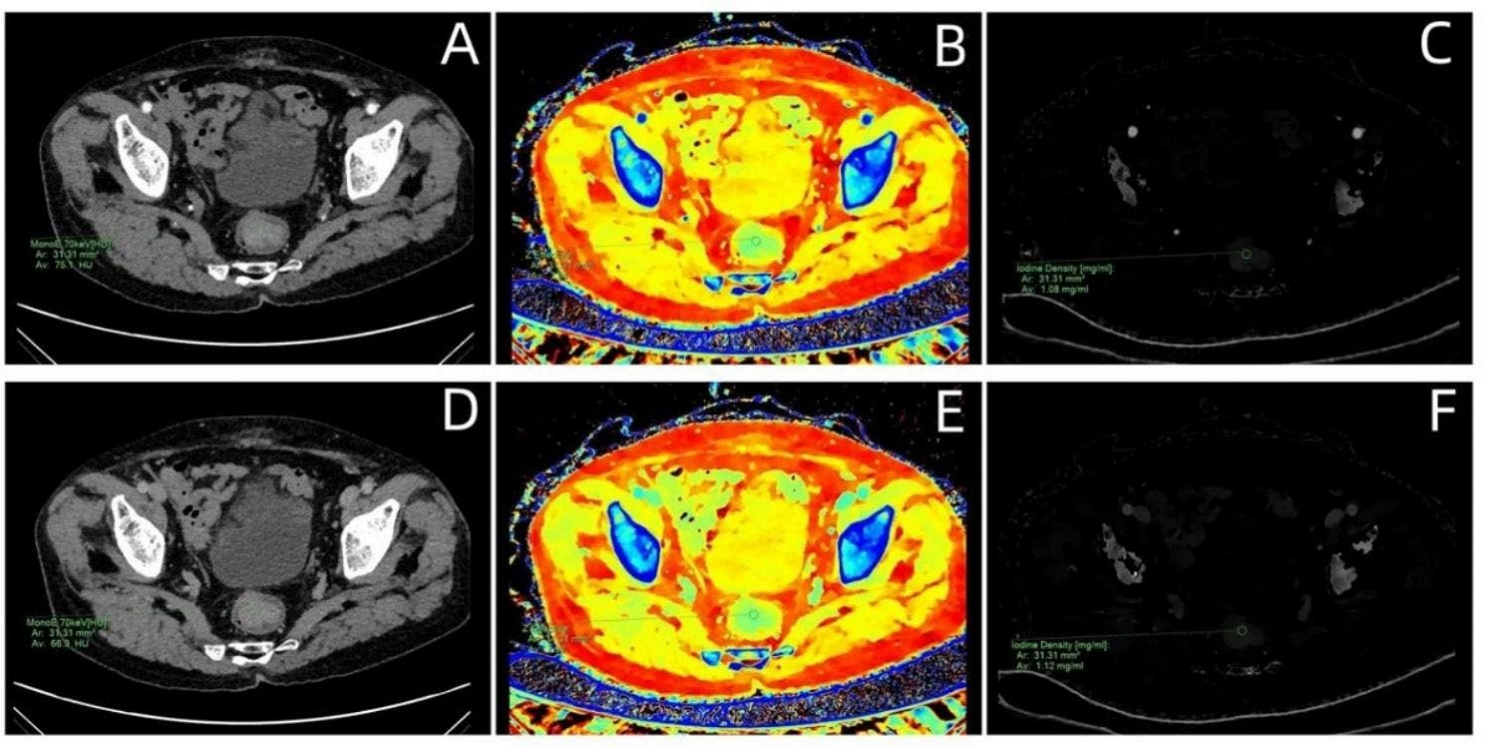
A man with colon cancer.DESCT parameters including monochromatic 70 CT value,iodine concentration (IC), and effective atomic number were measured with the same region of interest at the same location.The figure **[A-C]** shows the arterial phase,and the figure **[D-F]** shows the venous phase

The regions of interest (ROIs) were mapped independently by Two experienced radiologists who were unaware of the patient’s clinical and pathological data. Two radiologists delineated ROIs in 70 keV monochromatic images of section with the largest level of the tumor in arterial phase and venous phase which were avoided necrosis, blood vessels, cystic lesions and other non-solid parts. Philips IntelliSpace Portal (ISP) 7.0 was used to automatically calculate the IC value of tumor and abdominal aorta or iliac vessels, Zeff value and monochromatic CT value at each energy levels (40–90 keV). To minimize measurement bias, each value was measured three times and averaged as the final result. And NIC value is used uniformly to reduce the gap between patients. Formula is as follows. Examples of DESCT images with ROIs for evaluating quantitative measurements in patient with CRC are shown in Fig. 2.

1 $$NIC = \frac{{IC(leision)}}{{IC(artery)}}$$

### Intra group and inter group consistency analysis

A radiologist independently mapped the ROIs and measured values of all images, and then repeated the operation by randomly selected the images of 20 patients. The interval between the two was one month. Finally, another radiologist mapped the ROIs and measured values of the images of the same 20 patients. The intraclass correlation coefficient (ICC) was used to calculate within-group variation measured by one radiologist of two measurements and between-group variation measured by two radiologist. If the ICC value was less than 0.4, it was considered that the repeatability of diagnostic tests was poor; If the ICC value was greater than 0.75, then the repeatability of the diagnostic test was fairly good.

### Statistic analysis

SPSS25.0 statistic software was used for all statistic analysis and calculation. P＜0.05 on both sides was considered statistically significant. The quantitative variables of normal distribution were expressed as mean ± standard deviation (SD). The measurement data was tested to see if they conform to the normal distribution, if so, the two independent samples t-test was adopted and the homogeneity of variance was tested. Chi-square test was used to analyze the enumeration data.

## Results

### Patient’s characteristics

Basic clinicopathological and DESCT quantitative parameters information of 50 CRC patients is shown in Table 1 and Table 2. Among them, 16 were positive and 34 were negative for lymphovascular tumor thrombus. 23 were positive and 27 were negative for LN metastasis. Positive accounts of peripheral nerve invasion for a relatively small proportion. 8 was well differentiated and 42 were poorly differentiated.

**Table 1:** Clinicopathological Variables of CRC patients [mean ± SD or no. (%)]

DESCT characteristics	Lymphovascular Tumor Thrombus(+) (n = 34)	Lymphovascular Tumor Thrombus(-) (n = 34)	P-value	LN metastasis (+) (n = 23)	LN metastasis (−) (n = 27)	P-value	Peripheral nerve invasion(+) (n = 9)	Peripheral nerve invasion(−) (n = 41)	P-value	poorly differentiated	Well differentiated	P-value
NIC[a.u.]												
AP	0.17 ± 0.09	0.15 ± 0.06	0.423	0.15 ± 0.76	0.16 ± 0.73	0.433	0.14 ± 0.04	0.16 ± 0.80	0.376	0.16 ± 0.08	0.14 ± 0.43	0.594
VP	0.51 ± 0.13	0.43 ± 0.12	0.039	0.50 ± 0.11	0.46 ± 0.14	0.992	0.49 ± 0.13	0..45 ± 0.12	0.387	0.45 ± 0.12	0.50 ± 0.13	0.360
Eff-Z[a.u.]												
AP	0.76 ± 0.05	0.75 ± 0.04	0.546	0.75 ± 0.43	0.75 ± 0.49	0.750	0.75 ± 0.03	0.75 ± 0.05	0.674	0.75 ± 0.05	0.75 ± 0.04	0.898
VP	0.88 ± 0.18	0.90 ± 0.03	0.680	0.88 ± 0.14	0.91 ± 0.36	0.382	0.92 ± 0.03	0.89 ± 0.11	0.457	0.89 ± 0.11	0.91 ± 0.03	0.591
CT value [HU]												
40-AP	157.15 ± 35.03	152.26 ± 40.01	0.678	147.23 ± 36.19	159.45 ± 39.63	0.264	144.97 ± 37.93	155.77 ± 38.45	0.455	155.24 ± 38.81	146.40 ± 36.32	0.554
40-VP	175.61 ± 33.84	168.92 ± 34.42	0.523	169.24 ± 35.13	172.61 ± 33.66	0.731	170.83 ± 41.75	171.11 ± 32.71	0.983	169.14 ± 35.15	181.14 ± 27.04	0.366
70-AP	75.54 ± 14.62	75.21 ± 16.35	0.944	72.93 ± 15.96	77.34 ± 15.42	0.990	72.46 ± 16.82	75.94 ± 15.56	0.551	75.48 ± 15.71	74.46 ± 16.51	0.869
70-VP	83.60 ± 14.08	80.33 ± 14.09	0.448	79.38 ± 14.30	83.08 ± 13.83	0.928	79.64 ± 18.53	81.76 ± 13.10	0.687	81.35 ± 14.58	81.49 ± 11.47	0.981
90-AP	60.32 ± 11.60	61.28 ± 12.20	0.793	59.43 ± 12.32	62.29 ± 11.60	0.406	59.73 ± 11.52	61.25 ± 12.11	0.733	60.63 ± 12.20	62.82 ± 10.70	0.637
90-VP	66.07 ± 11.19	63.45 ± 11.67	0.457	62.29 ± 10.86	65.99 ± 11.90	0.260	62.23 ± 14.43	64.74 ± 10.88	0.558	64.62 ± 11.87	62.55 ± 9.59	0.645

### Interobserver Agreement

The ICCs of within-group variation for the IC values in the AP (ICAP), IC values in the VP (ICVP), Zeff in the AP (Zeff-AP), Zeff in the VP (Zeff-VP), 40-90keV monochromatic CT values in the AP (CTAP) and CT values in the VP (CTVP) were0.884, 0.710, 0.870, 0.674, 0.810, 0.531, 0.435, 0.737, 0.596, 0.440. And the ICCs of between-group variation for those were0.925, 0.836, O.909, 0.809, 0.897, 0.705, 0.627, 0,777, 0.538, 0.391. From the above data, we can draw a conclusion that the consistency of ICAP, Zeff-AP and 40kev-AP both in within-group variation and between-group variation were very well. Besides, the consistency of ICVP, Zeff-VP, and 70kev-VP in between-group variation were also excellent.

### Correlation of Clinicopathological Variables with Lymphovasucular Tumor Thrombus

68.7% of CRC patients who were positive for lymphovascular tumor thrombus were male, with a mean age of 63.00 ± 9.89 years. 69.6% of CRC patients who were positive for LN metastasis were male, with a mean age of 59.65 ± 10.58 years. 66.7% of CRC patients who were positive for peripheral nerve invasion were male, with a mean age of 56.11 ± 7.36 years. 66.7% of CRC patients who were poorly differentiated were male, with a mean age of 61.40 ± 11.20 years. And 62.5% of CRC patients who were Well differentiated were male, with a mean age of 64.50 ± 12.72 years. There was no statistical difference in age and gender between different groups(P≻0.05). (Table 1).

### Associations between DESCT Characteristics and Lymphovascular Tumor Thrombus

The NIC value of CRC patients who were positive for lymphovascular tumor thrombus were 0.17 ± 0.09 in AP, and 0.51 ± 0.13 in VP. The NIC value of CRC patients who were negative for lymphovascular tumor thrombus were 0.15 ± 0.06 in AP, and 0.43 ± 0.12 in VP. Table 2 showed that only NIC in VP was associated with lymphovascular tumor thrombus (p = 0.039),and other quantitative parameters were not.

**Table 2:** Clinicopathological Variables of CRC patients [mean ± SD or no. (%)]

Variables	Lymphovascular Tumor Thrombus(+) (n = 34)	Lymphovascular Tumor Thrombus(-) (n = 34)	P-value	LN metastasis (+) (n = 23)	LN metastasis (−) (n = 27)	P-value	Peripheral nerve invasion(+) (n = 9)	Peripheral nerve invasion(−) (n = 41)	P-value	Poorly Differentiated (n = 42)	Well Differentiated (n = 8)	P-value
Age(years)	63.00 ± 9.89	61.38 ± 12.11	0.644	59.65 ± 10.58	63.81 ± 11.87	0.200	56.11 ± 7.36	63.17 ± 11.77	0.092	61.40 ± 11.20	64.50 ± 12.72	0.486
Gender			0.778			0.623			0.963			0.820
male	11(68.7)	22(66.7)		16(69.6)	17(63.0)		6(66.7)	27(65.8)		28(66.7)	5(62.5)	
female	5(31.3)	11(33.3)		7(30.4)	10(37.0)		3(33.3)	14(34.2)		14(33.3)	3(37.5)	

The AUC value was 0.68. The ROC anaylisis showed that the optimal threshold value of NIC in VP value for diagnosis of lymphovascular tumor thrombus was 0.364. And the sensitivity was 68.8% and the specificity was 67.6%.

## Discussion

CRC is one of the most common gastrointestinal malignancies. The global incidence of CRC remains at a high level in recent years, which poses a serious threat to human health [13]. The early clinical symptoms of CRC patients are not typical, and most of them have developed into intermediate and advanced stages at the time of initial diagnosis, which seriously affects the prognosis of CRC patients.

The treatment plan and prognosis of CRC are closely related to histological type, differentiation degree and preoperative stage. At present,the main treatment for CRC is still surgical treatment, which is also the only treatment that can cure CRC. And the scope of surgical resection is closely related to tumor stage, so the accurate evaluation of tumor stage before operation is very important for the choice of surgical methods of CRC. At present,the most commonly used clinical staging method of CRC is the latest eighth edition of the American Joint Committee on Cancer (AJCC) Staging manual [14].

Lymphovascular tumor thrombus is defined as the thrombus formed by malignant tumor cells found in small blood vessels or small lymphatic vessels connected to the tumor [15], which means that the malignant tumor has invaded the mall blood vessels or small lymphatic vessels and is likely to have distant metastasis. In previous studies, lymphovascular tumor thrombus has been proved to be a pathological feature related to the prognosis of CRC [16]. The prognosis of CRC patients with lymphovascular tumor thrombus was significantly worse than that of negative patients [17].

For the moment, the most frequently used clinical detection method is HE staining. However, there are significant difference in the criteria of recognition, diagnosis and specimen treatment with lymphovascular tumor thrombus [18]. Therefore, although the traditional HE staining method can provide a large amount of diagnostic information, it still has many limitations, resulting in a lower detection rate of lymphovascular tumor thrombus.

Various non-invasive imaging examination have been used in the clinical practice of CRC, however, when CT and CT perfusion imaging are performed, patients are exposed to a large dose of radiation, and MRI and PET/CT are expensive and time-consuming, which are often unaffordable for most patients and cannot be widely applied in clinical practice. And MRI is difficult to distinguish between fibrosis and tumor invasion, which reduce the ability to differentiate early stage T3 and stage T2 tumors, physiological uptake of fluorodeoxyglucose by the gastrointestinal tract in PET/CT can lead to misunderstanding [19]. But DESCT not only has high image quality, but also has the function of multiple parameter and quantitative analysis. Now it has been used in the research of various diseases.

Iodine is the main component of contrast medium. Iodine concentration can reflect the blood flow of small blood vessels connected to the tumor and the lymph in the peripheral lymphatic vessels, so as to indirectly reflect the changes of tumor microcirculation and lymphatic circulation. The iodine concentration value can reflect the vessel permeability and density in the AP, and the iodine concentration can reflect the clearance rate and retention of contrast medium in the VP [20]. At present, there is no study using DESCT to predict the situation of lymphovascular tumor thrombus in patients with CRC. Therefore, we intend to use DESCT to evaluate the presence of lymphovascular tumor thrombus in patients with CRC.

The mean of the NIC value in the positive group was higher than that in the negative group of lymphovascular tumor thrombus in both venous and arterial stages in our study. The NIC-VP value of the positive group was 0.51 ± 0.13 while the NIC-VP value of the negative group was 0.43 ± 0.12, and the difference was statistically significant (P = 0.039). Therefore, it can be speculated that the NIC-VP value of CRC patients with lymphovascular tumor thrombus may be higher. It shows that the NIC value of DESCT can be used to evaluate the lymphovascular tumor thrombus of CRC. But the NIC-AP value of the positive group was 0.17 ± 0.09 while the NIC-AP value of the negative group was 0.15 ± 0.06, and the difference was not statistically significant (P = 0.423). We speculate that the reason may be due to some patients having early or delayed arterial phase scans, resulting in poor display of blood vessels. In addition, only 50 patients were included, which was a limitation for the results of this study.

## Conclusions

ROI region of interest sketch is to select a certain region of the tumor, which only represents the iodine concentration value of the selected region, and cannot accurately indicate the iodine concentration value of the whole tumor focus. At the same time, this study was carried out in a single-centre small numbers survey including some deficiency, so the multicentre large sample study was desired to establish. With the wide application of DESCT in clinical practice and study on large sample size, it is believed that DESCT can bring better help to clinical practice in the near future.

In conclusion, NIC value of DESCT has certain diagnostic significance for lymphovascular tumor thrombus in CRC patients, and DESCT is a more acceptable non-invasive examination method for patients, which can provide clinicians with more imaging information compared with conventional CT.

## Data Availability

The datasets used and/or analyzed during the current study are available from the corresponding author on reasonable request.

## Abbreviation

DESCT: dual-energy spectral computed tomography
CRC: colorectal cancer
HE: hematoxylin-eosin
NIC: normalized iodine concentration
Zeff: effective atomic number
AP: arterial phase
VP: venous phase
ROC: receiver operating characteristic
LNM: lymph node metastasis
ICC: intraclass correlation coefficient
